# P-1953. Youden’s Index Ensures the Accuracy of Wastewater-based Surveillance of SARS-CoV-2 Variants by Allele-Specific RT-qPCR or Genomic Sequencing

**DOI:** 10.1093/ofid/ofae631.2112

**Published:** 2025-01-29

**Authors:** Md Pervez Kabir, Elisabeth Mercier, Nada Hegazy, Tram B Nguyen, Chandler H Wong, Lakshmi Pisharody, Wan Shen, Patrick M D’Aoust, Graber Tyson, Robert Delatolla

**Affiliations:** University of Ottawa, Ottawa, Ontario, Canada; Univeristy of Ottawa, Ottawa, Ontario, Canada; University of Ottawa, Ottawa, Ontario, Canada; University of Ottawa, Ottawa, Ontario, Canada; University of Ottawa, Ottawa, Ontario, Canada; University of Ottawa, Ottawa, Ontario, Canada; University of Ottawa, Ottawa, Ontario, Canada; University of Ottawa, Ottawa, Ontario, Canada; Children's Hospital of Eastern Ontario Research Institute, Ottawa, Ontario, Canada; Univeristy of Ottawa, Ottawa, Ontario, Canada

## Abstract

**Background:**

Clinical genomic surveillance is the gold standard for monitoring SARS-CoV-2 variants globally, but as the pandemic wanes, reduced testing increases the risk of missing the emergence of variants of concern or failing to accurately follow their trajectory in populations. Wastewater-based genomic surveillance (WWS) that estimates variant frequency based on its defining set of alleles derived from clinical genomic surveillance has been successfully implemented. However, this method has its challenges, and allele-specific (AS) RT-qPCR that monitors a single, variant-defining, allele is used as a complementary method for estimating variant prevalence. Demonstrating equivalent performance of the methods is a prerequisite for their continued application of WWS in current and future pandemics.

**Figure 1:**
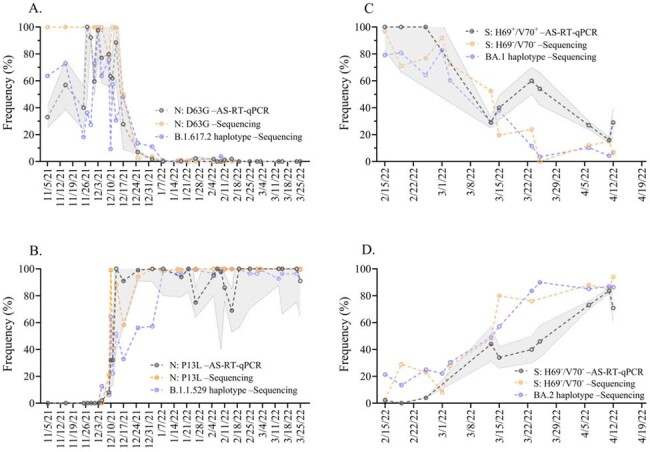
Comparison of AS-RT-qPCR and amplicon-based sequencing methods for estimating SARS-CoV-2 variants frequency in wastewaters; A) N: D63G and B.1.617.2 haplotype, B) N: P13L and B.1.1.529 haplotype, C) S: H69+/V70+ and BA.1 haplotype, and D) S: H69-/V70- and BA.2 haplotype.

**Methods:**

We compared single-allele frequency estimation using AS-RT-qPCR, to single-allele or haplotype frequency estimations derived from amplicon-based sequencing to estimate variant prevalence in municipal wastewater collected during emergent and prevalent periods of Delta, Omicron, and two of its sub-lineages in Ottawa, Canada.

**Table 1:**
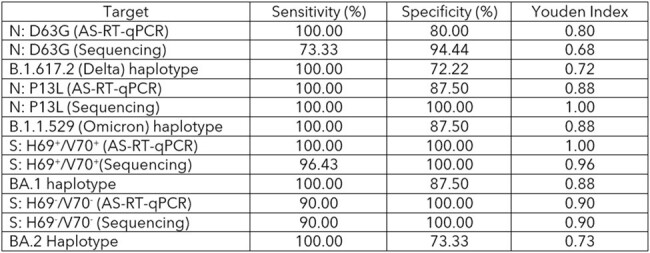
Youden index of targeted alleles (N: D63G, N: P13L, S: H69+/V70+ and S: H69-/V70-) in wastewaters using AS-RT-qPCR and sequencing as well as haplotype of each variant.

**Results:**

We found that all three methods of frequency estimation were concordant and contained sufficient information to describe the trajectory of variant prevalence in wastewater across time (Figure 1). We further evaluated the accuracy of these methods by quantifying the diagnostic performance (i.e., method accuracy expressed as Youden’s index), based on available clinical genomic prevalence data. Youden’s index confirms the accuracy of methods employed for variant frequency estimation in wastewater (Table 1), but accuracy of each method can be influenced by different factors.

**Conclusion:**

WWS emerges as a crucial epidemiological tool for monitoring infectious disease in the population during the COVID-19 pandemic. This study validates the accuracy of WWS in SARS-CoV-2 variants monitoring using AS-RT-qPCR or sequencing methods and provides comprehensive perspective for implementing public health interventions against infectious diseases.

**Disclosures:**

All Authors: No reported disclosures

